# Marginal Aggregates: The Role of Clays

**DOI:** 10.3390/ma17164153

**Published:** 2024-08-22

**Authors:** Arnon Bentur, Pavel Larianovsky

**Affiliations:** National Building Research Institute, Faculty of Civil and Environmental Engineering, Technion, Israel Institute of Technology, Haifa 3200003, Israel; lpavel@technion.ac.il

**Keywords:** marginal aggregates, micro-fines, clays, admixtures, clay mitigation

## Abstract

Clays are components in the fine portion of aggregates, less than 75 microns in size (micro-fines), which are usually washed away when producing coarse or fine (manufactured sand) aggregates in quarries. When marginal sources of aggregates are being used, the content of this washed portion can be quite high, and there is an incentive to keep as much of it in the aggregate, including the clays. The present paper presents a comprehensive treatment of the role of clays in terms of the characterization of their composition and quantification of their effects on the rheological and mechanical properties of cementitious systems, as well as the means to mitigate deleterious influences. It is shown that the strategy for neutralizing the effect of micro-fines containing clays on increased water demand in concrete can be quantified in terms of the combination of their content in concrete and their nature as characterized by the methylene blue value (MBV); this is a more rational approach to considering their influence than their content in specific aggregates as specified in standards. The effect of low and medium MBV aggregates on the water requirement can be neutralized by lignosulfonates when their content in the concrete is below a threshold value of about 150 kg/m^3^; polycarboxylates (PC) are required at higher contents; for high MBV aggregates, a combination of PC and clay mitigating admixture (CMA) is required. It is also demonstrated that with proper treatment, such micro-fines can be turned into useful fillers, enhancing the strength of concrete and thus also serving as a means for reducing cement content.

## 1. Introduction

The current environmental and ecological constraints lead to the need to extensively use marginal aggregates. As a result, there is interest in looking into the influence of fine materials smaller than 75 microns in size (micro-fines, or fines as termed in the EU standard EN 12620 [1]), which usually have been washed away during processing, with the intention of allowing incorporation of higher levels of them in the concrete, e.g., [2,3].

Several studies dealt separately with a range of issues, focusing mainly only on the standard methylene blue test [4,5,6,7], the influence of clays on the rheological behavior of mortars and concretes [8,9,10,11,12,13], and the mitigation of deleterious effects of clays by the use of admixtures [14,15,16]. All these issues are interrelated, and it was felt that there is a need for a comprehensive treatment encompassing all these aspects simultaneously in order to advance the fundamental knowledge base, including (i) the mineral composition of the clays, (ii) their characterization by standard test methods, (iii) their influence on rheological properties and their engineering quantification, (iv) the impact on hardened properties, and finally (v) the modifications by use of admixtures. An integrative project with this scope in mind has been carried out at the Technion [17,18].

This comprehensive treatment is required to deal with the gap that exists between the approach taken by the standards and the industry at large, viewing the clays as deleterious to concrete, while at the same time there is considerable advance in the field of admixtures, which may be mobilized towards enabling the use of aggregates with a higher content of clay. Bridging this gap is essential for enhancing the successful application of aggregates, which are currently considered marginal.

The approach taken by standards and specifications is to limit the clay content in aggregates by setting limiting maximum values for the methylene blue test, which provides indications to the content of clays and their nature (in particular swelling clays) in the aggregates. It is backed by studies that address the deleterious effects of clays in concrete, which are usually characterized by their influence on workability, strength, and dimensional stability [5,6,19,20,21,22]. The advent of methods to mitigate deleterious influences of clays in aggregates, e.g., [13,14,16,23], has not had an impact on specifications and standards. This is to a large extent the result of the traditional approach, which is focused on aggregate quality rather than concrete performance, where admixture technologies can be applied to mitigate the influence of concrete constituents and enable the use of marginal aggregates. The current paper is intended to provide a rational approach to meet the challenge in this gap, based on a systematic study of the influences of micro-fines on concrete performance in fresh and hardened states.

## 2. Materials and Methods

### 2.1. Overview of the Research Plan and Testing Program

The present report is based on a comprehensive study focusing on the evaluation of mortar and concrete systems in which clay containing micro-fines was incorporated with a size range of less than 75 microns.

The concept advanced, different from the current approach, is that focus should be given to the evaluation of the micro-fines since they are the portion of fine aggregate material (e.g., sand, smaller than 4.75 mm) that carries clays that might be deleterious to concrete. The overall methodology to deal with these effects should be based on an evaluation of the micro-fines quality in combination with their content in the concrete, rather than the sand quality as influenced by the clays in it. The final outcome, with respect to concrete standardization, should thus not be the sand quality but rather the micro-fines quality in combination with their content in the concrete. This should be the technological approach to mobilizing the use of marginal fine aggregate (sand) material. This approach is also consistent with the current initiatives to develop and design high performance concretes, such as UHPC (Ultra High Performance Concrete) and SCC (Self Consolidating Concrete), where micro-fines play a critical role. Mobilization of micro-fines, which are considered marginal, could also be useful for high-end applications.

The study consisted of two parts, the first of which was intended to characterize micro-fines derived from various processes in quarries and determine their effects on the behavior of mortars using a range of test methods, in particular rheological means. For that purpose, “engineered sand” samples were prepared, made up of various contents of micro-fines mixed with high quality siliceous sand.

The second part focused on the study of concretes in which micro-fines were incorporated and their fresh and hardened properties were characterized. The properties of the concretes were modified by the use of admixtures intended to mitigate the deleterious influences of the micro-fines. These included lignosulfonate (LS) and polycarboxylate (PC) to control the dispersion of the micro-fines, as well as clay mitigating admixture (CMA) to neutralize the influence of swelling clays. The concretes with optimal admixture type and content to control workability were also evaluated for strength and shrinkage.

The research strategy for both parts of the study was based on the concepts of preparing micro-fines of controlled compositions, which were obtained by using pure clay components and micro-fines recovered from by-products in quarries. In the testing of mortars and concretes, washed aggregates (sand and coarse aggregates) were used, which contained no micro-fines. Micro-fines were added directly to the mortar or concrete separately, and thus the aggregates used are referred to as “engineered”. The micro-fine characteristics are outlined below:

- Micro-fines recovered from quarries by-products were obtained by either taking material from clay rich lenses in quarries or fine waste materials in quarries and separating the micro-fines from them by a process that involved water washing and sieving through a 75 microns mesh, followed by drying to remove the water.
- The micro-fines obtained were characterized for their mineralogical composition by quantitative XRD and particle size distribution tests.
- For the characterization of the clay nature using the standard methylene blue test, which is adequate for aggregates containing clays, “engineered sand” was prepared. It consisted of 5% of the micro-fines mixed with 95% of high-quality siliceous sand, with particles smaller than 1 mm having a fineness modulus of 1. Thus, the Methylene Blue Value (MBV) for this engineered sand was used to characterize the clay nature of the micro-fines, which contained, in addition to clay, some other minerals, mainly Calcite, Dolomite and Quartz. The calibration curves of the MBV value against swelling clay, e.g., Montmorillonite, served as a way to provide the “equivalent Montmorillonite” content in the micro-fines.
- The mortars prepared and evaluated consisted of 1:3 cement:sand mixes. The cement was CEM I 52.5. The sand was “engineered” to contain the high-quality siliceous sand described above, in which portions were replaced by different micro-fine contents in the range of 0 to 30%.
- The concrete prepared consisted of washed aggregates containing no micro-fines. The micro-fines were added in controlled amounts, up to 300 kg/m^3^ in the concrete mix. To compare with the presence of micro-fines, which are “pure” fillers, high-quality calcium carbonate filler was used.
- The properties of the fresh mortars and concretes were characterized by rheological tests using a rotational ICAR viscometer for concrete and a ConTec rheometer for mortars. The characteristic value used in these tests was the yield value, which was shown to linearly correlate with the standard flow table test for mortars and the slump test for concrete.
- The properties of the mature mixes evaluated were strength and drying shrinkage.

### 2.2. Characterization of Micro-Fines

The micro-fine mineralogical composition was evaluated by XRD, their clay content and type were characterized by the methylene blue test using the ASTM C 1777 procedure [24], and their particle size distribution was determined by a laser-type instrument in which a dispersion solution was employed.

A systematic evaluation of the effects of these micro-fines containing clays was carried out by investigating the fresh and hardened properties of 1:3 cement:sand mortars in which the sand was “engineered”, being composed of high quality siliceous sand with a fineness modulus of 1, and replacing portions of it with fine material with a size smaller than 75 microns. This micro-fine material was either pure clay minerals, Kaolinite and Montmorillonite, or the fine materials obtained from the clay lenses in quarries, which were rich in clay but contained other minerals as well, such as Calcite, Dolomite and Quartz. This procedure provided mixes with varying contents and types of clays.

The composition of the micro-fine material obtained from the quarry clay lenses was determined by XRD, and the quantitative mineral composition was estimated by analysis using the Rietveld method. The physical properties of these fines were characterized by the ASTM D 4318 test [25], which is used for soils. The parameters quantified were the liquid limit, plasticity limit, and plasticity index.

The clay nature of the micro-fines was determined by the methylene blue test using the ASTM C 1777 procedure. This test is based on the quantification of the adsorption of methylene blue by the tested material using the colorimetric method. For that test, which is intended for sand, the “engineered sand” outlined above was prepared, consisting of 95% high quality siliceous sand with 5% of the micro-fines to be characterized.

The properties of the mortar mixes in the fresh state were evaluated by means of a rheological test to determine the yield stress and apparent viscosity values, as well as by the flow table test using the ASTM C 1437 [26] and ASTM C 230 procedures [27] to determine the spread and flow values. These tests were carried out with mixes of different w/c ratios. The compressive strength of these mixes under wet curing for 1, 7, and 28 days was also determined.

A special series of tests using these methodologies was carried out in order to characterize the effect and effectiveness of different admixtures, including conventional water reducing admixtures, lignosulfonates, and PC, as well as CMA and Clarena, a product of WR Grace.

### 2.3. Micro-Fines Mineralogical Composition and Particle Size Distribution

The mineralogical composition of “pure” components (calcium carbonate, Montmorillonite, and Kaolinite clays) and micro-fines is presented in Table 1.

**Figure 1:**
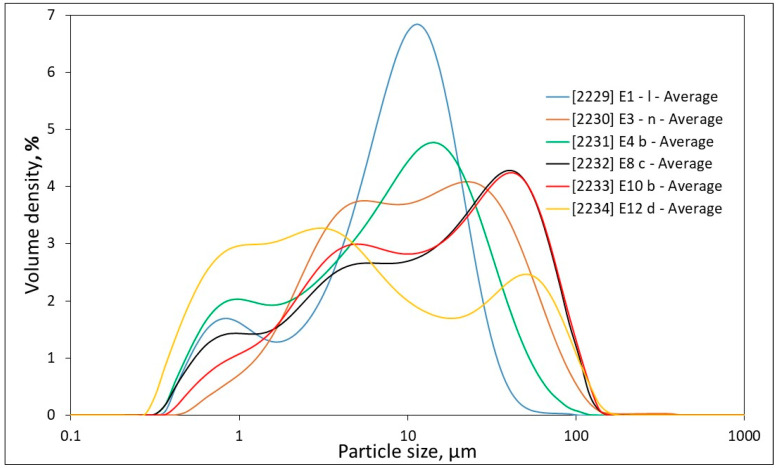
Particle size distributions of the micro-fines (notations as in Table 1).

The particle size distribution data are presented in Table 2 and Figure 1. For comparison, the data also includes calcium carbonate, which was used in this study as a reference for “pure” filler in concrete. There is a drastic difference between the nature of the particle size distributions of the micro-fines, which is much wider, relative to the calcium carbonate, which is more of a mono-size distribution. The table also includes the clay content determined by the XRD test and the MBV values determined by the methylene blue test (see Section 2.2), which provide a quantitative indication for the presence of swelling clays, in particular Montmorillonite. This test is very sensitive to the presence of clays, which are usually in small amounts, a few percent or less, and can thus detect clays that are not always readily detected by the quantitative XRD.

### 2.4. The Methylene Blue Test

**Figure 2:**
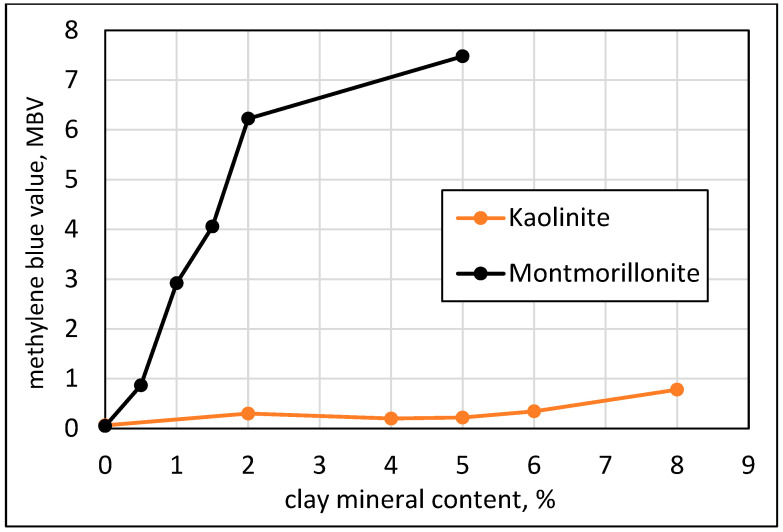
Relations between methylene blue value and the content of clay minerals in “engineered sand” samples with “pure” clay minerals, Kaolinite and Montmorillonite, using the ASTM C 1777 procedure with 20 g sample.

Since the work here was intended to quantify the effects of clays in terms of their content and type, calibration runs of the methylene blue (MB) test were carried out to determine the Methylene Blue Value (MBV) as defined by ASTM C 1777. Calibration curves of “engineered sand” samples with varying content of pure clay minerals are presented in Figure 2.

The curve is very shallow for the Kaolinite, demonstrating that the adsorption capacity of the methylene blue is extremely low. For the Montmorillonite, there is a steep linear relationship up to about 2%, reaching a relatively high value of MBV of 6, and thereafter the increase is mild. The high slope up to 2% of the Montmorillonite is indicative of the high adsorption capacity of this clay compound. The mild increase afterwards might be interpreted as an art effect, which is due to the fact that the 0.5% concentration of the methylene blue solution dictated by the standard is already adsorbed with 2% Montmorillonite; an increase in the content of this mineral beyond the 2% value cannot be accompanied by a marked change in the adsorbed solution.

It can be shown by a simple calculation, taking into consideration the 0.5% concentration of the solution, its content of 30 g, and the 20 g quantity of sand mixed with it, that the maximum methylene blue value that can be reached under these conditions is 7.5. This is the value approached in Figure 2 with less than 5% Montmorillonite. The implication is that if higher concentrations of Montmorillonite are expected in the sand, the test procedure should be modified to enable higher levels of adsorption by reducing the quantity of the tested sand. Indeed, the standard recommends using a smaller amount of the tested material (10 g instead of 20 g) when the methylene blue value is approaching 7.5 to account for this effect. The data in Figure 2 suggest that this change should take place even at a lower methylene blue value of about 6. In the following sections, the methylene blue values obtained by the two procedures, the standard with 20 g and the one recommended for high values of methylene blue using 10 g samples, will be reported. They will be addressed as the 20 g and 10 g procedures.

## 3. Results

### 3.1. Methylene Blue Values

**Figure 3:**
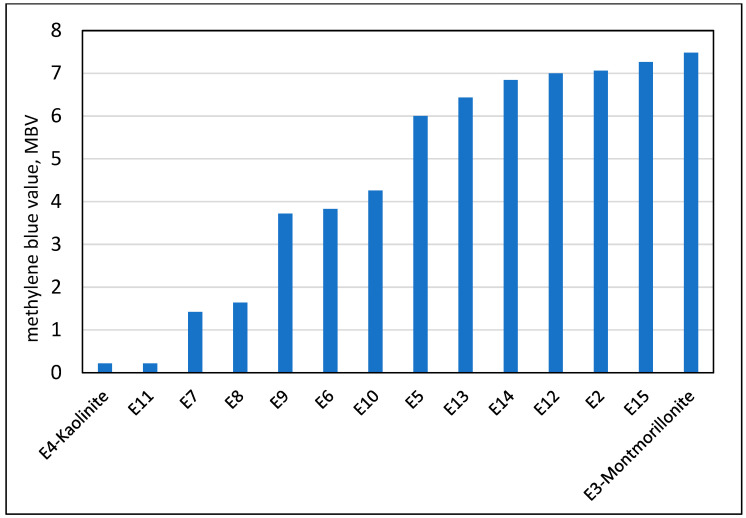
The methylene blue values (20 g procedure) of engineered sands containing 5% of micro-fine (<75 micron) clay type material, either pure minerals or material obtained from clay lenses in quarries.

The results of the characterization of the micro-fine material of the quarry lenses using the MBV values of “engineered sand” containing 5% of the micro-fines and 95% of high-quality siliceous sand are shown in Figure 3. It also includes engineered sands with 5% of the pure clay minerals, Kaolinite and Montmorillonite. The data in Figure 3 are for materials obtained from different quarries in Israel, and it is sorted by the increased level of the MBV.

It can be seen that the lower bound is engineered sand with Kaolinite and the upper bound is with Montmorillonite, almost 7.5. In between, there is a range of values from about 0.2 to slightly more than 7 of the sands engineered from the quarry lenses.

### 3.2. Mineralogical Composition

The Rietveld quantitative mineralogical analysis based on the XRD patterns confirmed that the pure Kaolinite and Montmorillonite were indeed pure, having contents of about 95% of the relevant mineral. The mineralogical analysis of the clay micro-fine material obtained from the quarry lenses showed a variable composition consisting of Kaolinite, Montmorillonite, Calcite, Dolomite, and Quartz, with the clay minerals being present in significant quantities, between 20 and 60% (Table 1).

**Figure 4:**
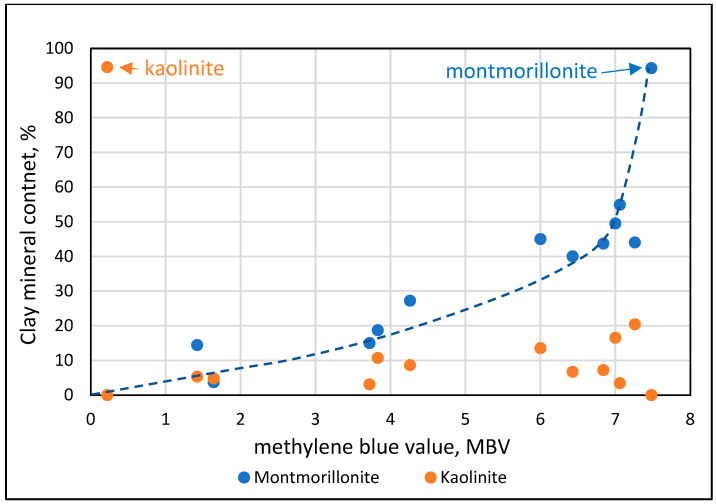
Content of the clay minerals Kaolinite and Montmorillonite as a function of the methylene blue values (20 g test procedure) of the engineered sands containing 5% of micro-fines, either pure clay minerals or <75 micron material obtained from clay lenses in quarries.

The plot of the clay mineral content in the engineered sands as a function of the MBV value, Figure 4, highlights some of the mineralogical variables and their relations with the MBV value.

**Figure 5:**
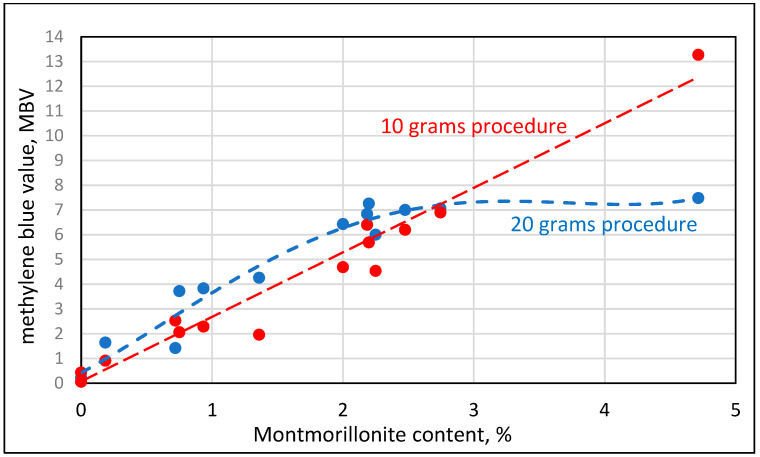
Relation between the methylene blue value obtained by the 20 g and 10 g procedures and the Montmorillonite content in engineered sands containing 5% of micro-fines, either pure clay minerals or material obtained from clay lenses in quarries.

The pure clay engineered sands have similar contents of the relevant minerals, Kaolinite and Montmorillonite, 5% of the total sand, but their methylene blue value (MBV) is drastically different, very low for Kaolinite, 0.22, and very high for Montmorillonite, 7.48. All the other engineered sand samples fell in between. Their MBV could be correlated with an increase in their Montmorillonite content but not with changes in the Kaolinite content. The very steep increase in the Montmorillonite–MBV relation, starting at an MBV of about 6, is a reflection of the reduced sensitivity of the methylene blue test at this range when it is carried out according to the ASTM procedure with 20 g of material. This is demonstrated when plotting the MBV values of the 20 g procedure as a function of the Montmorillonite content, Figure 5, showing a plateau for the “20 g procedure” curve when exceeding the contents of about 2% Montmorillonite. The increase in the Montmorillonite content above 2% should have been accompanied by an increase in the uptake of methylene blue, but this does not take place as almost all of the methylene blue in the 0.5% solution is apparently adsorbed at about 2% of Montmorillonite. When plotting the relation between the Montmorillonite content and the MBV obtained by the 10 g procedure, a linear relation is obtained with R^2^ = 0.96, demonstrating the significant correlation between the MBV value and the mineralogical composition, as shown in Figure 5 (“10 g procedure” curve). A comparison of the two curves in Figure 5 suggests that when the MBV value in the standard test procedure with 20 g exceeds about 6, the procedure with 10 g is the one that should be employed, as it better reflects the changes in the mineralogical composition.

### 3.3. Rheological Behavior Characterization

**Figure 6:**
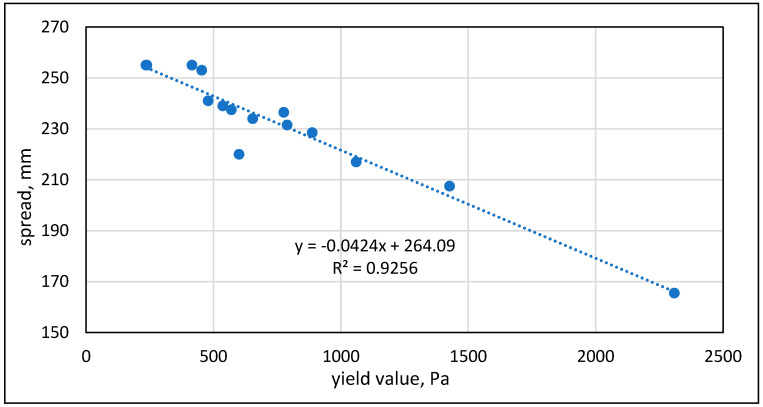
Relations between the yield value obtained in the rheological test and the spread obtained in the ASTM flow table test of mortars with 0.92 w/c ratio.

The rheological behavior was characterized in terms of the yield value, which was found to correlate quite well with the spread obtained in the ASTM flow table test for mortars (Figure 6).

**Figure 7:**
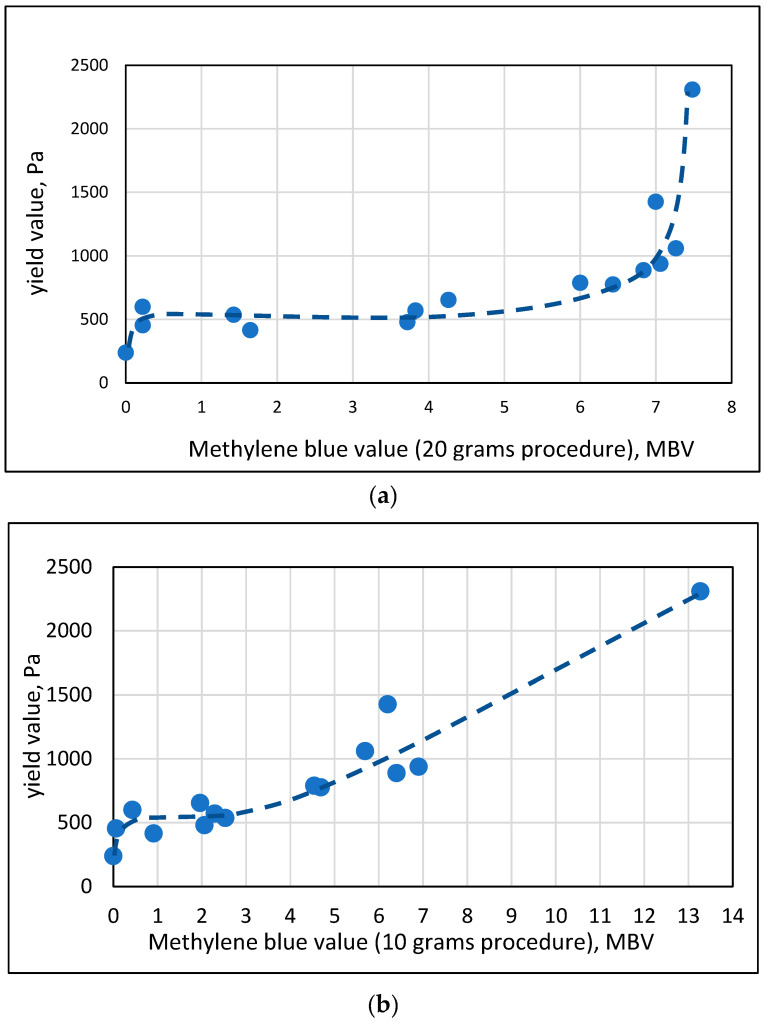
Relation between the yield value and the methylene blue value (**a**) 20 g procedure and (**b**) 10 g procedure for mortars of 0.92 w/c ratio with engineered sands containing 5% of micro-fines, either pure clay minerals or material obtained from clay lenses in quarries.

The effect of the nature of the micro-fines in the engineered sands was quantified for mortars with a 0.92 w/c ratio. This high w/c ratio was chosen for comparison since, at this w/c level, a wide range of micro-fines could be investigated, ranging from ones with a very high yield value (small spread) to ones with a very low yield value (high spread) without the need to use flow enhancing admixtures. The specimens investigated exhibited differences of more than one order of magnitude in the yield values, and these were presented as a function of the MBV of the engineered sands, Figure 7, for the MBV obtained by the 20 g and 10 g procedures.

At the lower range of the MBV, there is a doubling of the yield value once 5% of the sand is replaced with Kaolinite, as the MBV value increases from 0 (for the straight sand) to 0.22 for the Kaolinite. At the high range, the yield value increases by more than an order of magnitude when 5% of the sand is replaced with Montmorillonite. Between these two extremes, there seems to be a plateau in the yield value up to about MBV of 3. Thereafter, there is an increase, monotonic for the 10 g procedure (Figure 7b) and a very mild one for the 20 g procedure, in the range of MBV of 3 to 6. Afterwards, a very steep increase takes place towards an MBV of 7.5. Obviously, the trend in the 10 g procedure is a better reflection of the nature of the effect of the MBV value.

**Figure 8:**
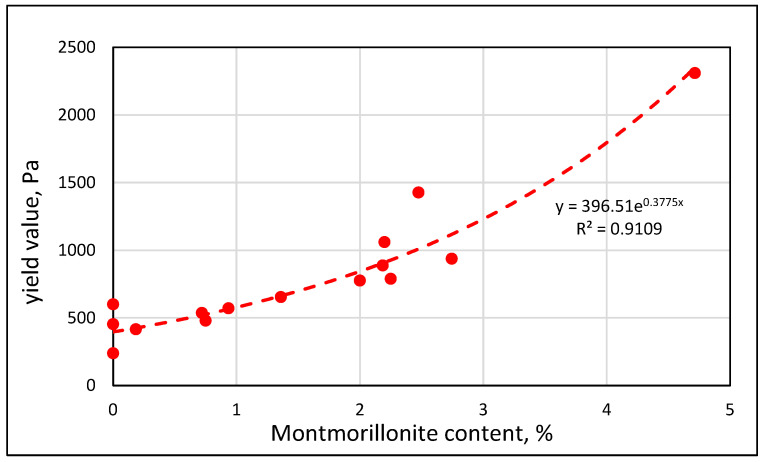
Relation between the yield value and the Montmorillonite content in mortars of 0.92 w/c ratio with engineered sands containing 5% of micro-fines, either pure clay minerals or material obtained from clay lenses in quarries.

One may try to interpret the changes in the slope of the yield value vs. MBV curves in terms of different mechanisms. If one plots the yield value as a function of the Montmorillonite content, Figure 8, a continuous increasing curve is observed, following an exponential relationship. The nature of this relationship is such that in the range of up to about 1% Montmorillonite, the change in the yield value is relatively small, and beyond that, it increases markedly with the increase in the Montmorillonite content. This trend is consistent with the observations in Figure 7 that, up to an MBV value of about 3, the change in the yield value is relatively small. It may be interpreted in terms of two mechanisms: one dominant up to 1% Montmorillonite, which is perhaps less affected by the adsorption capacity of the clay, and the other above 1% Montmorillonite, where the adsorption influence of the Montmorillonite is becoming much more dominant.

### 3.4. Characterization of Micro-Fines by Plasticity Tests

**Figure 9:**
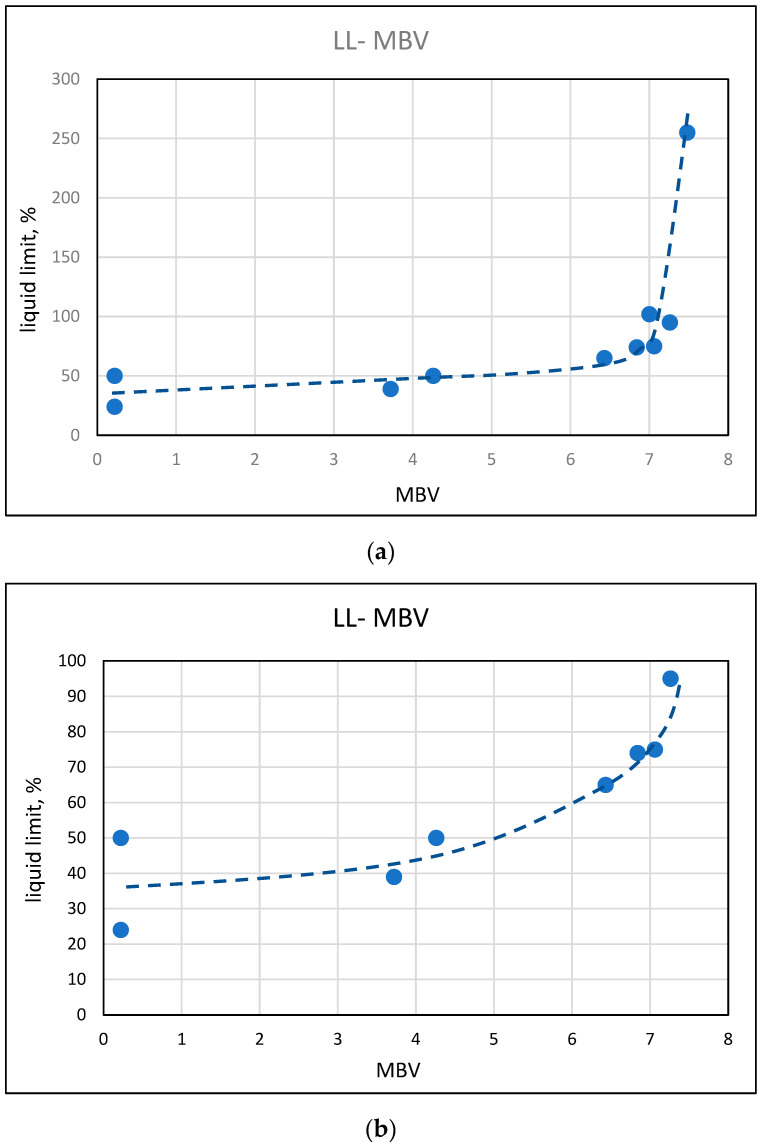
Relationship between the liquid limit and the methylene blue value (**a**) in the range up to 300 liquid limits and (**b**) in the range up to 100 liquid limit.

**Figure 10:**
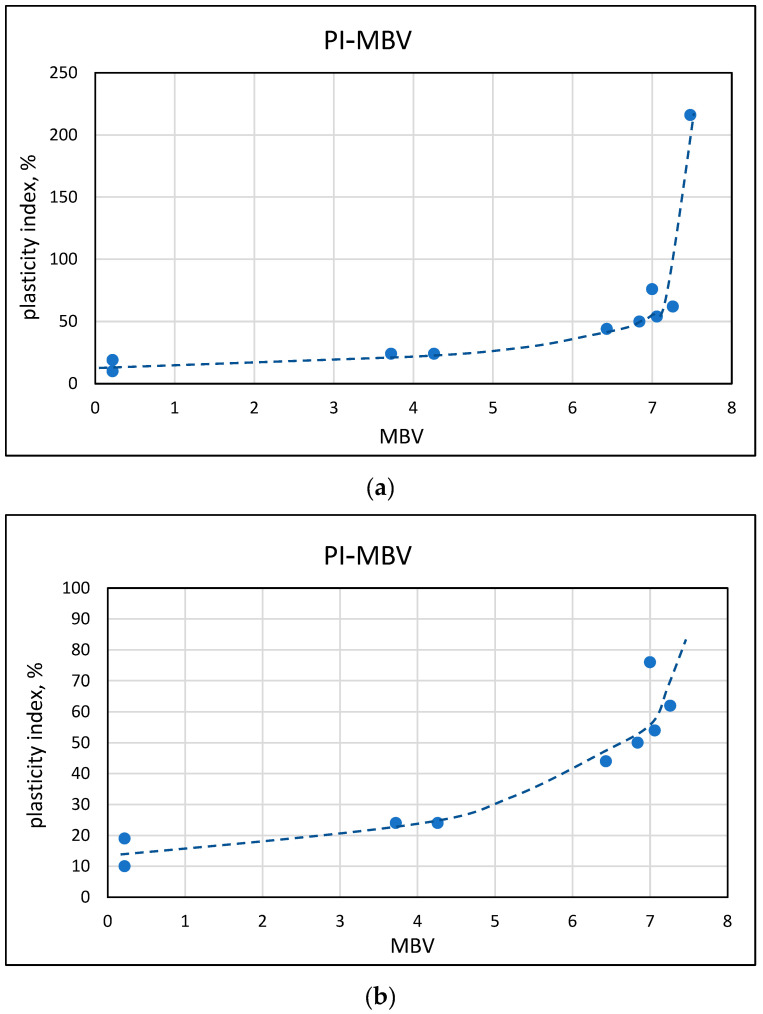
Relationship between the plasticity index and the methylene blue value (**a**) in the range up to 250 plasticity index and (**b**) in the range up to 100 plasticity index.

The results of the plasticity tests, liquid limit, and plasticity index, which are common for soils, are presented in Figure 9 and Figure 10 as a function of the MBV values. The trends resemble those observed in Section 3.3, with regards to the effects taking place at different ranges of MBV. Up to values of about 3 to 4, there is a very mild increase in the plasticity parameters; thereafter, a more marked exponential increase is being observed, which becomes very steep at methylene blue values in the range of 6 to 7.

## 4. Discussion

The mortar part of the study was intended to provide a simulation for the concrete. Its purpose was to explore the influence of the clays in the macro-fines on the behavior of the fresh system in order to define several categories of micro-fines quality. The concept is that for each category, a different strategy for admixture control should be applied to mitigate detrimental influences. Three such categories were established. The effectiveness of this classification was verified for concretes, in which micro-fines of the different categories were incorporated at the expense of the sand. The verification dealt with influences on the fresh and hardened properties of the concrete.

### 4.1. Effects of Admixtures

**Figure 11:**
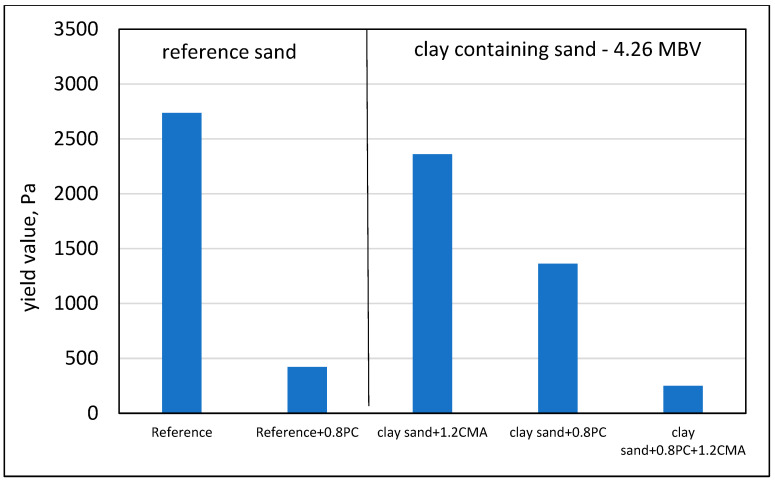
The yield values of 0.70 w/c ratio mortars with reference sand (MBV of 0) and with engineered sands (MBV of 4.26), as influenced by the presence of 0.8% ploycarboxylate admixture (PC), 1.2% clay mitigating admixture (CMA), and combination of these two.

The obvious means to neutralize the detrimental influence of the clay containing sand on the rheological behavior is the use of admixtures. A series of tests was carried out using different types of admixtures and their combinations, evaluating their influence in w/c ratio mortars of 0.92 and 0.70 w/c. The lower w/c ratio mortar is a better simulation of the mortar phase in normal strength concrete. In particular, the influence of PC admixtures and CMA, which adsorb into the clay surfaces, was studied. Typical results are presented in Figure 11, comparing the influence of the admixtures in mortars with the reference sand and in mortars with engineered sand having an MBV value of 4.26 (sand containing 1.36% Montmorillonite and 0.43% Kaolinite). The comparison in Figure 11 is for 0.8% by weight of PC, with and without 1.2% of CMA.

It can be seen that the 0.8% PC is very efficient in reducing the yield value in the reference sand, yet in the clay containing sand, the yield value still remains much higher with the same content of PC. The effect of the CMA seems to be rather small when it is on its own, as the yield value remains rather high in the clay containing sand mortar. However, the combined influence of the PC and CMA in the clay containing sand seems to be very significant, suggesting perhaps a synergistic effect in which the CMA is expected to neutralize the adsorbing effect of the clay, thus allowing the full effectiveness of the PC by presumably preventing its adsorption by the clay that contains Montmorillonite.

### 4.2. Mortar Rheological Behavior

As outlined in Section 2.1, the approach to be taken is a comprehensive one, considering at the same time the quality of the micro-fines, as estimated by the MBV, and their content in mortars. This should be the basis for developing the admixture strategy to mitigate detrimental influences, rather than looking at the sand’s overall behavior. This strategy could enable us to mobilize the positive influences of the micro-fines in high end applications to generate filler effects.

**Figure 12:**
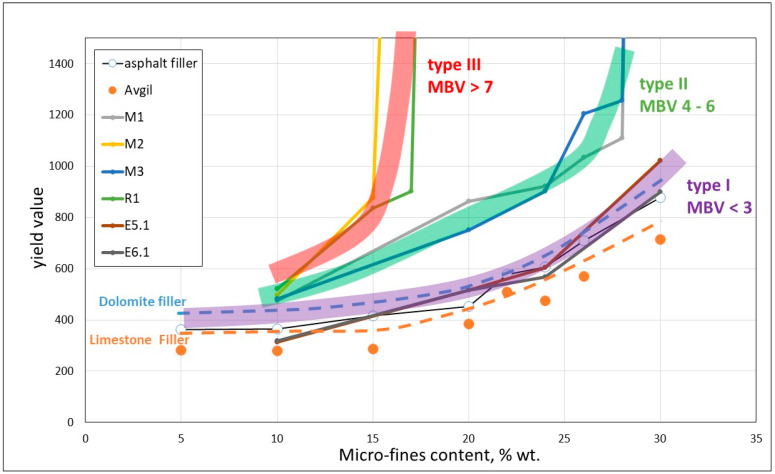
Relations between yield value and micro-fines content in engineered sand mortars for different levels of MBV.

The underlying hypothesis of this study [28] was that the effect of the micro-fines should be evaluated based on a combined effect of their nature (i.e., content and type of clay) and their total content in the concrete rather than in a specific aggregate (usually sand) as currently addressed in standards. To verify this approach, all the data were presented in terms of the relation between the yield value of mortars prepared from engineered sands with varying contents of micro-fines of different natures, up to 30% content of the sand, and their content in the engineered sand, Figure 12. The data in Figure 12 can be characterized by three categories of families of curves: Type I with an MBV smaller than 3, Type II with an MBV between 4 and 6, and Type III with an MBV bigger than 7. It should be emphasized that the MBV of the micro-fines is determined on the basis of the MBV of an engineered sand consisting of 5% of the micro-fines with 95% of high quality siliceous fine sand.

The difference between these types of families of curves is in the initial slope of the curves and the micro-fines content at which the slope tends to shift to much higher values, taking place at about 30% for Type I, 25% for Type II, and 15% for Type III.

**Figure 13:**
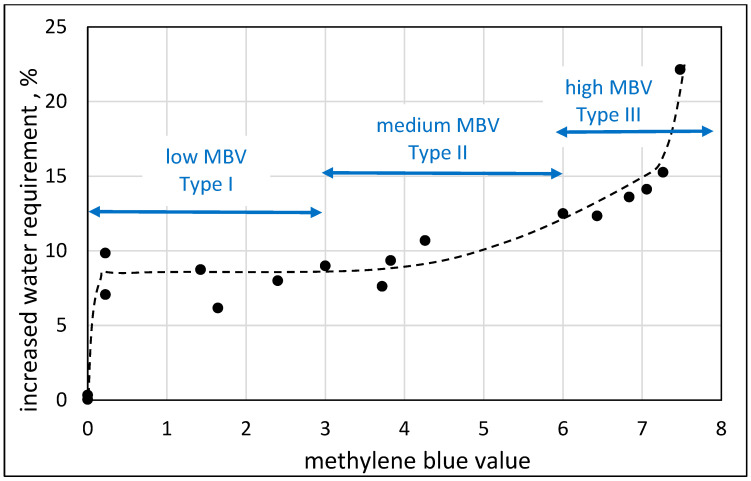
The effect of the methylene blue value of the engineered sand and the increase in water demand in 0.7 w/c ratio mortars made from this sand.

The differences in nature between the three types of curves also show up in the quantification of their influences on the water demand of mortars. This is demonstrated in Figure 13 for 0.70 w/c ratio 1:3 cement:sand mortars, with engineered sand containing 20% of micro-fines of different natures (quantified by the MBV value). The differences between the categories are quantified by their influence on the increase in water demand: (i) up to 8% for type I; (ii) a gradual increase with MBV from about 8% to 13% for Type II; and (iii) a marked increase with MBV for Type III.

### 4.3. Concrete Workability Control

The different nature of the three types of families in Figure 12, with respect to their influence on the increase in water demand, suggests that various admixture strategies need to be adjusted to mitigate these influences. These include water reducing admixtures for Types I and II, either lignosulfonates or PC, whereas for Type III, there is also a need to combine CMA.

To study the implication of these three types in concrete, three samples of micro-fines, characterized by differences in their MBV values, were used to be in the range outlined in Figure 13: Type I (MBV in the range smaller than 3), Type II (MBV in the range of 4 to 6), and Type III (MBV bigger than 7). They were derived from by-products in quarries by a procedure of separating the micro-fines through a 75 microns mesh. The three were different in their composition, in particular in the equivalent content of Montmorillonite, as calculated from MBV tests. The mineralogical composition, in addition to the clays, consisted mainly of Dolomite and Calcite, as shown in Table 3, which also includes a Dolomite micro-fine (filler). All these micro-fines served for the preparation of concrete with various quantities of micro-fines and a range of admixtures. The admixture content and type were adjusted to obtain similar workability at a given w/c ratio of 0.75. This concrete composition yields a grade of 30 MPa using CEM I 52.5 cement. Workability was characterized by slumps and yield values.

The concrete base mix composition is outlined in Table 4. The effect of the micro-fines was evaluated by studying mixes in which 75 to 300 kg/m^3^ of micro-fines of different qualities (MBV values) which were incorporated at the expense of the sand. In all the mixes, the contents and combination of admixtures (L—Lignosulfonate, PC—Polycarboxylate, and CMA—Clay Mitigating Admixture) were adjusted to keep the same workability (about 200 mm slump).

**Figure 14:**
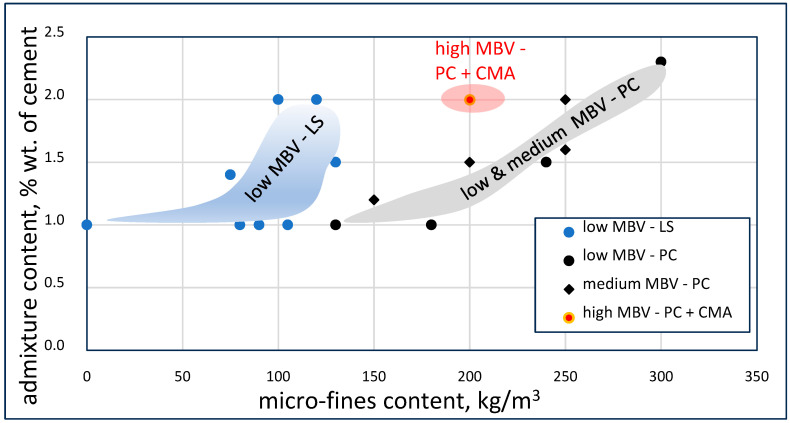
The types of admixtures and admixture combinations required to maintain workability in concretes containing different types (MBV values) and contents of micro-fines.

The test results were presented in terms of a diagram showing the effect of the micro-fines content and quality (quantified by their MBV) on the content and type of admixture required to maintain the workability (slump and yield value) of the reference concrete having no micro-fines. The total content and the MBV of the micro-fine aggregate serve as guidance for the admixture strategy required, as shown in Figure 14.

The diagram in Figure 14 can serve as a demonstration for the strategy suggested in this paper to deal with clays in concrete aggregates by considering the micro-fine content and nature. It calls for considering the effect of the micro-fines in the concrete at large rather than their presence in the various aggregate fractions. It can provide guidelines for the mix design with respect to the type and content of admixtures that need to be incorporated. It shows that for low and medium MBV values of the micro-fines, Lignosulfantes can be sufficient to mitigate deleterious effects on workability, up to about 150 kg/m^3^ of micro-fines. Above that value, the content of the Lignosulfonates required would approach 1.5–2.0%, which is the range where they cause retardation, and therefore there is a need to resort to PCs. The PCs could be effective even at levels of 300 kg/m^3^ of micro-fines, as long as the micro-fines are of Type I and II (low and medium MBV). If, however, the micro-fines are of type III, there is a need for a combination of PC and CMA if the micro-fines content is 200 kg/m^3^.

The results of this study also indicate that the quantity of CMA required is related to the equivalent Montmorillonite content in the clay. It is estimated that the CMA content should be approximately 1/3 of the Montmorillonite content in the micro-fines in the concrete. The equivalent content of the Montmorillonite can be estimated based on the MBV using the calibration curves, such as those presented in Figure 5.

### 4.4. Concrete Mature Properties: Strength and Shrinkage

**Figure 15:**
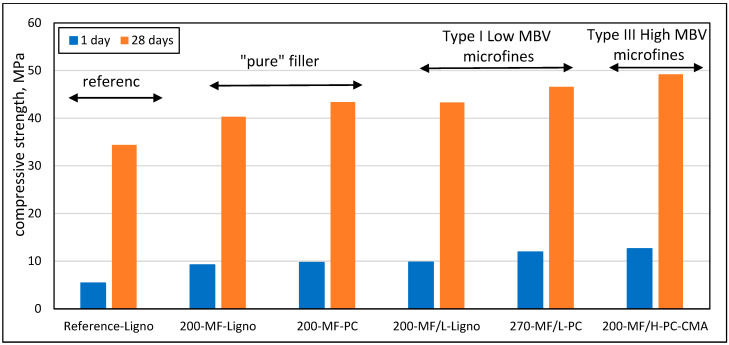
Effect of content and type of micro-fines, in combination with admixtures to maintain constant w/c ratio and workability, on concrete strength at 1 and 28 days; the numerical values indicate the micro-fines contents in kg/m^3^ units.

The strength tests in this study indicate that if the effect of clays on workability is mitigated by the proper admixture strategy, filler effects could be mobilized, as shown by the increase in strength accompanying the presence of micro-fines, Figure 15. The figure shows that for 200 kg/m^3^ of micro-fines in concrete, a 28 days strength increase could be observed, up to about 30%, with higher values obtained with Type II and III micro-fines. This enhanced value for the Type II and III micro-fines suggests that the clays possess added value in addition to the Carbonate and Dolomite particles in the micro-fines. This trend was even more evident at one day.

**Figure 16:**
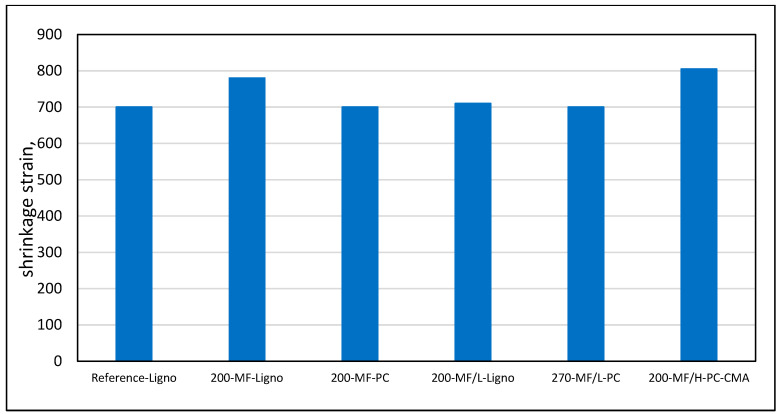
Effect of content and type of micro-fines in combination with admixtures to maintain constant w/c ratio and workability on concrete after 90 days of drying shrinkage; the numerical values indicate the micro-fines and filler contents in kg/m^3^ units.

Shrinkage tests demonstrated that at the optimum admixture contents, the shrinkage was practically independent of the micro-fines content, Figure 16. This is a further support for the hypothesis that when the micro-fines are properly dispersed and the detrimental effects of swelling clays in them are neutralized, they act as fillers rather than detrimental “dust” as was assumed in the past.

## 5. Summary and Conclusions

Clays are components in the fine portion of aggregates, less than 75 microns in size, which is usually washed away when producing coarse or fine (manufactured sand) aggregates. When marginal sources of aggregates are being used, this washed portion can be quite high, and there is an incentive to keep as much of it in the aggregate, as is reflected in current standards, in which the allowed content of micro-fines has increased in recent years. In order to avoid deleterious effects of clays that might remain in these micro-fines, the standards have adopted limitations on the clay content that are based on the MBV value. There is thus significance for the appraisal of the nature of quantification obtained by the MBV test and the scope of influences that should be considered in attempting to use aggregates with higher levels of clay. Some of the issues and conclusions highlighted in this study are outlined below:

- The deleterious influence of clays on their rheological properties is largely due to the presence of swelling clays like Montmorillonite, whereas Kaolinite has only a small effect. This difference in behavior can be attributed to the swelling and water uptake characteristic of Montmorillonite. The methylene blue test is indicative of this influence.
- The current study indicates that when the clay content, and in particular the Montmorillonite component in it, is above about 2%, the MBV value obtained by the standard 20 g procedure, which is about 6, is not sufficiently sensitive to quantify the presence and influence of the clay. There is thus a need to resort to the 10 g procedure.
- There is potential for using high clay content aggregates based on a strategy of utilizing admixtures. Yet, for that to be effective, there is a need to develop formulations of blends of admixtures that should be adjusted for the composition and content of the clay.
- These concepts were mobilized for providing guidance on the use of high contents of micro-fines containing clays in concrete, as summarized below:

1. Micro-fines can be categorized into three types, depending on their MBV; low (less than 3), medium (4–6), and high (more than 7), showing differences in increased water demand in each category.
2. The strategy for neutralizing the effect of micro-fines on increased water demand in concrete can be quantified in terms of the combination of their content in concrete and their MBV; this is a more rational approach to considering their influence than their content in specific aggregates as specified in standards.
3. The effect of low and medium MBV aggregates on the water requirement can be neutralized by lignosulfonates when their content in the concrete is below a threshold value of about 150 kg/m^3^; PC is required at higher contents; for high MBV aggregates, a combination of PC and CMA is required.
4. At the optimum admixture content and type, high micro-fines content in aggregates can provide a beneficial effect on strength, suggesting that the filler effect is being mobilized without having a detrimental effect on shrinkage. This beneficial effect can be mobilized to reduce cement contents in concrete.

## Figures and Tables

**Table 1:** Mineralogical composition and MBV values of micro-fines.

	E3 *	E4 **	E2	E5	E6	E7	E8	E9	E10	E11	E12	E13	E14	E15
Quartz		2.1	39.9	38.2	8.1	6.2	23.2	6.2	21.4	0.8	15.2	37.2	58.8	40.7
Christobalite	51.6													
Calcite			32.6	9.1	67.1	11.0	9.8	60.9	55.8	45.0	22.0	46.1	13.0	7.7
Dolomite			1.3	25.9	12.9	66.6	37.6	7.9	1.8	14.4			4.7	8.2
Ankerite	2.5						4.7	18.2	4.2	39.8	55.3	6.2		5.8
Rutile			2.1	2.3									3.8	
Anhydrite			0.7	0.6									1.5	
Montmorillonite (clay)	45.9		6.3	1.7	0.3		0.3	0.3	1.1		6.5	3.4	6.7	
Kaolinite (clay)		84.6	7.4	10.7	9.4	1.8	19.8	5.4	14.1		20.7	7.0	5.6	18.5
Halloysite (clay)			9.7	11.5	1.4	14.4	4.7	1.2	1.5				6.0	19.1
Illite (clay)		6.7												
Muscovite (clay)		6.7												

* Source for high content Bentonite, technical grade. ** Source for high content Kaolinite, technical grade.

**Table 2:** Statistical parameters of the particle size distribution of the micro-fines and their mineralogical nature with respect to the presence of clays.

Notation	Nature of Sample	D (10%) µm	D (50%) µm	D (90%) µm	<2 µm %wt.	<8 µm %wt.	Clay Content, XRD, %wt.	MBV
E1	Limestone	1.03	7.89	19.41	16.5	50.6	0.0	0.00
E3	Montmorillonite	2.09	10.30	43.58	9.3	43.2	94.3	7.48
E4	Kaolinite	0.91	7.77	27.92	21.2	50.9	94.0	0.22
E8	Micro-fines of sand	1.17	12.83	56.41	16.0	40.6	8.5	1.64
E10	Micro-fines of sand	1.68	12.99	57.71	21.4	40.0	35.8	5.26
E12	Micro-fines of sand	0.66	3.98	50.72	33.9	63.4	66.0	7.00

**Table 3:** Characterization of the four types of micro-fines.

Micro-Fine Type	MBV 20% Dilution	MBV 30% Dilution	Montmorillonite Equivalent Content, % wt.	Dolomite, % wt.	Calcite, % wt.
“Clean” (R)	0	0	0	98.6	1.4
Low MBV(E37)	3.0	4.6	5.5	88.8	5.8
Medium MBV (E27)	4.4	5.6	8.0	82.5	9.5
High (E)	7.2	10.5	13.7 (11.0 by XRD)	79.5	6.8

**Table 4:** Base concrete mix composition.

Content, kg/m^3^				
Cement	Coarse Aggregate	Graded Sand	Sand	Water
330	645	745	337	250

## Data Availability

The original contributions presented in the study are included in the article, further inquiries can be directed to the corresponding author.
